# A Connection Between the Gut Microbiome and Epigenetic Modification in Age-Related Cancer: A Narrative Review

**DOI:** 10.14336/AD.2024.1618

**Published:** 2026-01-20

**Authors:** Florida Owens, Joseph Souchak, Valeria Nazaire, Juliet Akkaoui, Rajib Shil, Candy Carbajal, Kingshuk Panda, David Caraballo Delgado, Inge Claassen, Santiago Moreno, Samantha Yi, Yishu Dong, Nirbachita Adrita, Lee-Seng Lau, Nazira El-Hage

**Affiliations:** Department of Cellular and Molecular Medicine, Herbert Wertheim College of Medicine, Florida International University, Miami, Florida, 33199, USA.

**Keywords:** aging, gut microbiome, polypharmacy, cancer, epigenetic

## Abstract

As individuals age, physiological changes influence the composition and function of the gut microbiome, significantly impacting the onset and progression of various illnesses, including cancer. Notably, the gut microbiome affects epigenetic modifications such as DNA methylation and histone alterations. Furthermore, it contributes to the age-related decline in immune system efficiency, increasing susceptibility to infections and cancers. This dual role of the gut microbiome—both a protective factor and a risk factor—is a key aspect of its importance in maintaining long-term health, making it a significant topic of discussion in this review. Moreover, a challenge faced by the elderly is the concurrent use of multiple medications. Polypharmacy can interact with the gut microbiome, potentially altering its efficacy, leading to adverse drug reactions, and affecting vital microbiome diversity. The effects of these interactions on cancer therapies and the overall health of elderly patients are becoming increasingly important. Understanding the complex relationship between aging, the gut microbiome, cancer, and polypharmacy is crucial for developing more effective therapeutic strategies and improving patient outcomes. Here, we discuss recent advances in understanding age-related physiological changes in the microbiome and their significance in cancer development and therapy. Specifically, we will explor how epigenetic changes acquired during aging, along with ongoing prescriptions of multiple medications and the decline of immune function, contribute to the intricate relationship between aging and cancer.

## INTRODUCTION

As life expectancies increase, so does the number of individuals living with age-related chronic conditions that impact daily activities. Aging is defined as the functional and persistent decline of physiological functions crucial for survival and reproduction throughout a lifetime. It is characterized by a gradual buildup of molecular and cellular damage over time and the physical limit of cell division due to telomere shortening. Telomere attrition leads cells to enter a state known as senescence, a limitation referred to as the Hayflick limit [1-4]. This process is driven by clearly defined hallmarks of aging, which include DNA instability, epigenetic alterations, mitochondrial dysfunction, loss of proteostasis, exhaustion of stem cells (or loss of stemness), deregulated nutrient sensing, cellular senescence, altered intercellular communication (such as chronic inflammation and age-associated dysbiosis), and impaired macroautophagy (cellular self-digestion) [3-5]. Gut microbiota and surrounding host tissue have emerged as important regulatory factors that can become functionally mature as early as three years old, influencing physiological processes and disease development, including cancer, throughout a person's lifetime [6, 7]. As people age, they face a higher risk of developing multiple diseases (comorbidities), which leads to an increased likelihood of being prescribed numerous medications, culminating in a phenomenon known as polypharmacy [8, 9]. Patients with cancer are frequently prescribed numerous medications for both the disease and supportive care, which places them at an increased risk of medication-related incidents [10, 11].

Cancer is marked by the abnormal growth of cells that have lost their ability to regulate growth and divide uncontrollably [12-14]. The hallmarks of cancer include genomic instability and mutations, the acquired ability to sustain proliferative signaling, resist cell death, activate invasion and metastasis, evade growth suppressors, enhance vasculature, permit replicative immortality, deregulate cellular metabolism, promote tumor inflammation, avoid immune destruction, unlock phenotypic plasticity, interact with polymorphic microbiomes, undergo non-mutational epigenetic reprogramming, and accumulate senescent cells [13, 15, 16]. The risk of developing cancer increases significantly with age. According to the National Cancer Institute, the average age of individuals diagnosed with cancer is 66 years. The incidence of cancer rises with age until around 85 years old, after which it quickly declines as a primary cause of mortality to less than 5% [17-19]. However, the numbers can vary depending on the type of cancer. For instance, according to the National Cancer Institute, the median age at diagnosis for breast cancer is 62 years; for colorectal cancer, it is 67 years; for lung cancer, it is 71 years; and for prostate cancer, it is 66 years. Some cancers diagnosed during childhood or adolescence occur without signs of accelerated aging or the simultaneous development of other age-related conditions [20]. However, they will not be addressed in this review.

Although aging and cancer may seem like different processes, they share common characteristics since cellular damage typically leads to the development of both conditions [3]. The rise in the aging population is contributing to an unprecedented increase in cancer cases and fatalities worldwide, reinforcing the connection between these two issues. Age-associated pathologies, such as inflammation, involve uncontrolled cell growth or hyperactivity, which are also linked to cancer. This raises speculation that certain hallmarks of aging, or their mechanistic drivers, may promote oncogenesis and tumor progression while others may inhibit carcinogenesis.

This review discusses recent advancements in understanding age-related physiological changes in the microbiome and their relevance to cancer development and therapy. Specifically, we examine how epigenetic changes acquired during aging, combined with the ongoing use of multiple medications and a decline in immune function, contribute to the complex relationship between aging and cancer.

### (i) AGE-RELATED CANCERS AND THE GUT MICROBIOME

#### The Gut Microbiome in Health and Disease

The gut microbiome is a key factor in developing and progressing numerous age-related diseases and chronic non-communicable diseases. Although aging is unavoidable, its damaging effects on physical and cognitive functions are not experienced evenly. The microbiome has a complex relationship with age; it changes as the host ages and is modified by age-related diseases, while also influencing the severity of age-related impairments in the host. Personal factors such as progressive physiological decline and lifestyle habits like diet, exercise, medication, and reduced social interaction induce changes in the gut microbiome [21]. The significance of these factors is evident in the shifting global demographics. The United Nations forecasts that within the next 30 years, the population aged 65 and older will more than double, reaching 1.5 billion worldwide, with most residing in developing countries [22]. As a result, healthcare costs will rise significantly unless the determinants of healthy aging are thoroughly addressed. Emerging data links changes in the microbiome to an increasing number of diseases, including inflammatory bowel disease, liver diseases, obesity, diabetes, cardiovascular disease, neurological dysfunction, and gastrointestinal cancers such as pancreatic, colorectal, and prostate cancer [23-40].

**Figure 1.**
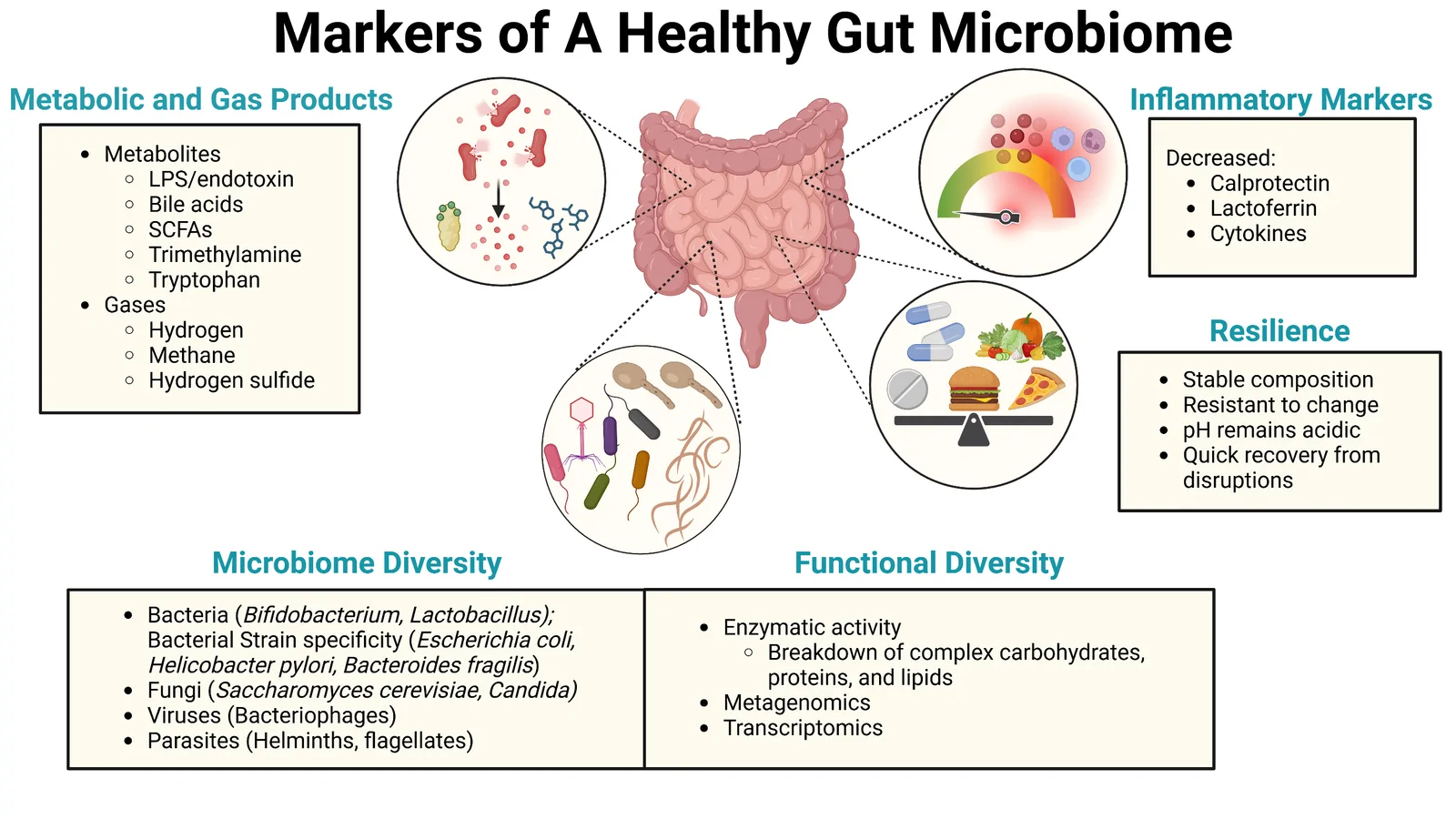
**Markers of a healthy gut microbiome**. Analyses of the human gut microbiome have found that a high volume of microbes and abundant but distinct microbial strains can carry out a diverse range of enzymatic and functional activities by producing various metabolites and gases. Altogether, these factors are associated with a low inflammatory response, promoting a healthy, functional microbiome resilient to perturbations.

There are several indicators of healthy gut microbiota (Fig. 1), including high bacterial diversity, bacterial strain specificity (*Escherichia coli, Helicobacter pylori, Bacteroides fragilis*), functional diversity, referring to the range of functions performed by the microbiota, including metabolic products produced by microbiota such as short-chain fatty acids (SCFAs), bile acids, and tryptophan [41, 42]. It also encompasses the production of various gases by microbiota like hydrogen, methane, and hydrogen sulfide, pH levels ranging from 5.5 to 7, and inflammation markers (e.g. calprotectin and lactoferrin)—where low levels of inflammatory markers in the stool indicate a healthy gut [43-52]. Furthermore, the gut microbiota's ability to be resilient and maintain a stable composition while resisting disturbances is essential [53]. However, no strict criteria still define what constitutes a healthy gut microbiota. Each marker has advantages and limitations, and the complex interplay between these factors adds to the challenge of objectively defining gut health [54]. Determining what constitutes a healthy gut microbiota has been an essential focus of recent research. While connections between an imbalanced or dysbiosis gut can be recognized, establishing causation and predicting disease development based on microbiome composition is complex.

With a resident community of approximately 2,000 bacterial species, regulating the gut microbiota environment involves a complex interaction between gut tissue and bacteria [55, 56]. These microorganisms' genomes are called microbial genomes, containing over 150 times the number of genes found in the human genome [57]. Consequently, the microbiome is considered to possess its genome. Microorganisms can coexist with host cells at various body sites, and these factors interact to influence the host's physiological and pathological processes [58-61]. As previously mentioned, a healthy gut microbiome includes a diverse array of microorganisms, such as beneficial bacteria (e.g., *Bifidobacterium, Lactobacillus*), fungi (e.g., *Saccharomyces cerevisiae*, Candida), viruses (e.g., Bacteriophages), and parasites (e.g., helminths, flagellates), all contributing to the maintenance of intestinal mucosal integrity, nutrient and drug metabolism, vitamin synthesis, immune system balance, digestion, and overall health [62, 63].

#### The Gut Microbiome and Aging

Throughout human life, the gut microbiome exhibits some predictable patterns, beginning with rapid changes from infancy to age three, stabilizing until middle age, and undergoing accelerated changes starting in late adulthood. According to numerous studies of centenarians from various countries, their gut microbiome composition is characterized by a lower abundance of health-related symbionts found in younger age groups (such as *Faecalibacterium* spp.) and an elevated abundance of both alternative health-associated taxa (such as *Akkermansia* spp.) as well as disease-associated pathobionts [64]. These general aging-related changes are characterized by a loss of dominant commensal taxa such as *Prevotella*, *Faecalibacterium*, *Eubacterium rectale*, *Lachnospira*, *Coprococcus*, and the health-associated genus *Bifidobacterium* [65-67]. These taxa appeared to be replaced by a second group of commensals such as the putatively beneficial *Akkermansia*, *Christensenellaceae*, *Butyricimonas*, *Odoribacter*, and *Butyricicoccus* and pathobionts such as *Eggerthella*, *Bilophila*, Fusobacteria, *Streptococcus*, and *Enterobacteriaceae*) [66, 67]. A third group of commensal microbial markers, such as *Akkermansia*, *Odoribacter*, *Butyricimonas*, *Butyrivibrio*, *Oscillospira*, *Christensenellaceae*, and *Barnesiellaceae* was shown to increase with unhealthy aging. It is essential to maintain a healthy gut microbiome by consuming a diverse range of plant-based foods, such as fruits (e.g., bananas, apples), vegetables (e.g., mushrooms, spinach), whole grains (e.g., barley, quinoa), and legumes (e.g., beans, lentils). Eating prebiotic fibers (e.g., oats) and probiotic-rich foods (e.g., yogurt) can also positively influence the gut microbiome [68-70]. A high-salt diet can change the gut microbiome and worsen metabolic disorders like hypertension and obesity [71].

#### Gut Microbiome Dysbiosis and Cancer Development

The risk of developing cancer rises with age because of a combination of genetic mutations, hormonal changes, immune system decline, and cumulative environmental exposures [72-82]. Age-related cancers, including pancreatic, breast, ovarian, prostate, colorectal, and lung cancer, rank among the most common in older adults. Understanding the specific risks and mechanisms contributing to their development is crucial for enhancing early detection, prevention, and treatment strategies for aging populations. Predictions for 2030 indicate that the prevalence and mortality associated with age-related cancers are expected to increase by over 60% in the aging population [83-87]. Although few microbes directly cause cancer, many seem to play a role in its growth and development, often influencing the host’s immune system [88]. Gut dysbiosis contributes to the development and progression of age-related cancers due to the vital role of microbiota in regulating inflammation, metabolism, and immune system function. Reducing microbial diversity results in hormonal changes and low-grade chronic inflammation, known as inflammaging, a hallmark of age-related cancers [89-91]. The dysfunctional immune system, influenced by a changing gut microbiome, involves elevated myeloid-derived suppressor cells (MDSCs) that deactivate antitumor immune responses, increased expression of pro-inflammatory cytokines (interleukin-1α/β, interleukin-6 (IL-6), and tumor necrosis factor-alpha (TNFα), and monocytes and macrophages that promote processes such as angiogenesis, enhancing tumor growth and metastasis, as well as weakened dendritic cell function, which impairs the antitumor T-cell response [92-94]. Aging neutrophils and macrophages weaken, reducing their ability to fight pathogens and amplifying tumorigenesis and inflammation caused by senescent cells. These mechanisms create a microenvironment that fosters tumorigenesis, aiding tumor cells' growth, survival, and migration while undermining immune defenses and cellular homeostasis [95].

#### Characterizing the Gut Microbiome in Age-related Cancers

**Figure 2.**
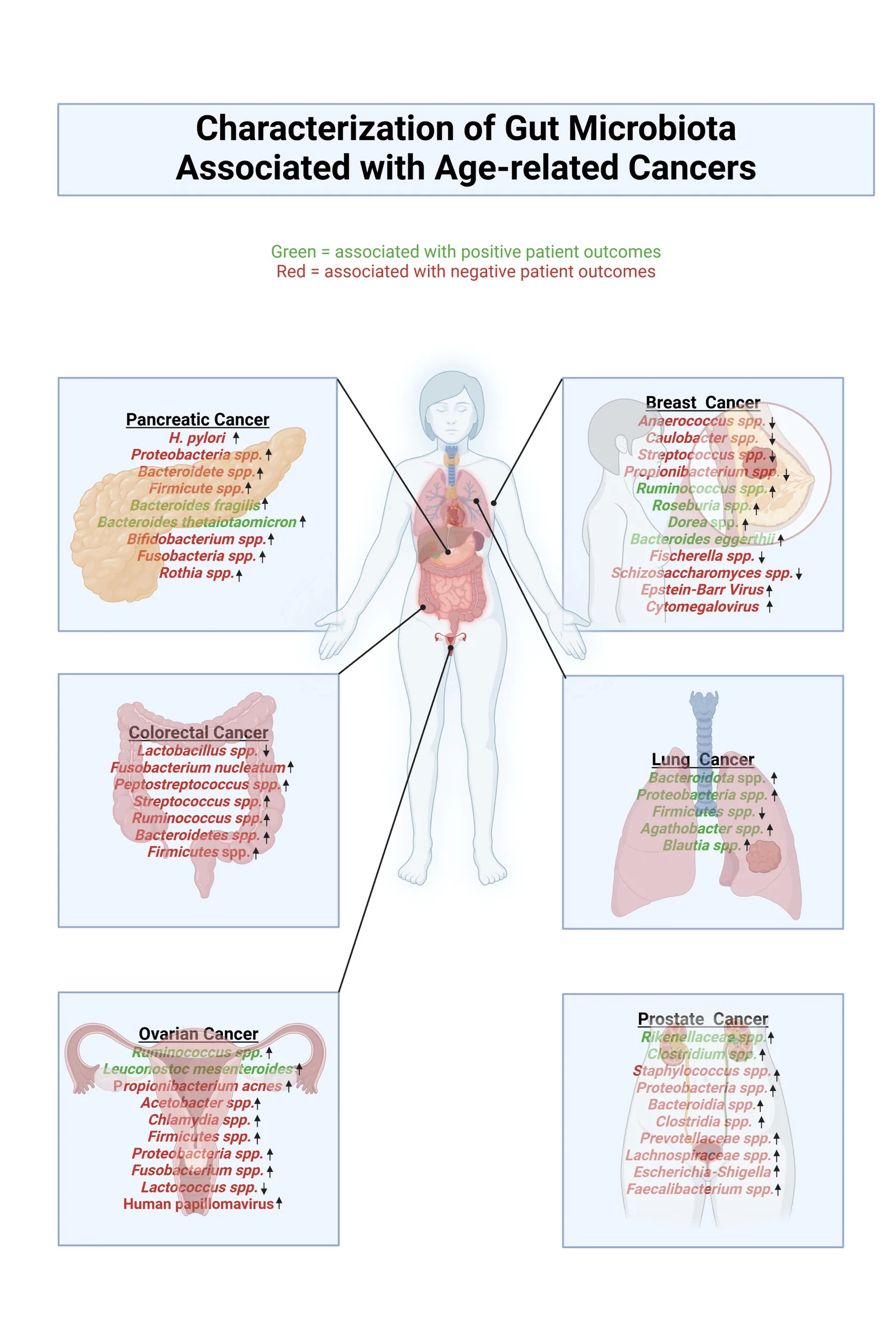
**Characterization of Gut Microbiome in Age-Related Cancers**. Recent studies have implicated many bacterial species and some viruses in the occurrence, growth, and prognosis of various age-related cancers.

Using 16S rRNA and 18S rRNA gene sequencing along with polymerase chain reaction (PCR) techniques, multiple researchers have begun to characterize the intratumoral microbiota in various tumors (Fig. 2), and there is evidence indicating that intratumoral bacteria correlate with patient prognosis [96]. In pancreatic cancer (PC), the bacterial composition of the tumor differs from that of a healthy pancreas [97]. *Helicobacter pylori* DNA has been found in the pancreas of 75% of PC patients, and an increasing number of studies have detected *Proteobacteria*, *Bacteroidete*, and *Firmicute* species were also abundant in PC patients [96, 98, 99]. Pancreatic ductal adenocarcinoma (PDAC) is among the leading causes of tumor-related deaths and the most common malignant neoplasm of the pancreas [100]. Metabolomic analysis of serum samples from PDAC patients who responded to cancer treatment, those who did not respond, and mice transplanted with microbiota from these groups identified indole-3-acetic acid (3-IAA) as the most enriched metabolite in the group that responded to cancer treatment [101, 102]. 3-IAA is a tryptophan metabolite derived from amino acid fermentation in the gut, driven by *Bacteroidetes* and *Firmicutes* species. *Bacteroides fragilis* and *Bacteroides thetaiotaomicron in particular* were more prevalent in the microbiota of respondent patients than nonrespondent patients [101]. A case-control study utilizing 16S and 18S to compare bacterial and fungal profiles of duodenal fluid collections from patients undergoing duodenal endoscopy, with and without PDAC, revealed reduced microbial diversity in patients over 70 years of age, as well as in patients with PDAC, compared to age-matched controls [103]. There was evidence of enrichment of *Bifidobacterium, Fusobacteria*, and *Rothia* bacteria among patients with PDAC with short-term survival [103]. Other microbial species unique to PC also include *Enterococcaceae, Lactobacillaceae, Nakaseomyces, Skeletocutis, Porphyromonas, Prevotella, Ruminococcaceae, Corynebacterium, Escherichia, Propionibacterium*, and *Streptococcus* [104-107].

Intestinal flora imbalances significantly contribute to colorectal cancer (CRC) progression. CRC prediction models and patient cohorts strongly associate age-related dysbiosis with a decline in healthy bacteria (e.g. *Lactobacillus*) alongside elevated levels of bacteria that contribute to chronic inflammation, including *Fusobacterium, Peptostreptococcus, Streptococcus, Ruminococcus, Bacteroidetes*, and *Firmicutes*, which contribute to the stimulation of tumor growth [108-110]. The mechanism of *Fusobacterium nucleatum* in CRC is well known, as it promotes the inhibition of immune response, modulates the severity of cancer, regulates microRNA (miRNA) and long noncoding RNA (lncRNA), and alters metabolic processes like autophagy [111-113]. Analysis of the gut microbiome from baseline fecal samples of colorectal cancer patients using 16S rRNA gene sequencing revealed a significantly increased relative abundance and positive detection rate of *Fusobacterium* in patients who do not respond to chemotherapy than those whose bodies react positively to treatment. Non-responders with high levels of Fusobacterium had shorter progression-free survival rates (the time during and after treatment that a patient lives with the disease without it worsening) than those with low microbe levels [114]. Mouse xenografts of primary human colorectal adenocarcinomas retained viable *Fusobacterium*, and treatment with the antibiotic metronidazole reduced *Fusobacterium* load, cancer cell proliferation, and overall tumor growth [115]. These compelling results call for further exploration of antimicrobial interventions as a potential adjunctive therapy for patients with microbial-associated colorectal issues and cancer.

The gut microbiome is correlated with the responsiveness to chemoradiotherapy in patients with non-small cell lung cancer (NSCLC). Patients with NSCLC in two phase 2 clinical trials (NCT02573506 and NCT03006575) were analyzed, and the amount of *Bacteroidota* and *Proteobacteria* increased, while the amount of *Firmicutes* decreased after concurrent chemoradiotherapy [116]. Patients with long progression-free survival exhibited elevated fatty acid metabolism, fatty acid biosynthesis, and arginine biosynthesis compared to those with short progression-free survival, who showed increased lipopolysaccharide (LPS) biosynthesis, ascorbate and aldarate metabolism, and the biosynthesis of vancomycin group antibiotics [116]. Gut microbiota was also found to correlate with antitumor activity in the single-arm phase II CAVE-mCRC and CAVE-LUNG clinical trial patients who had metastatic colorectal cancer (mCRC) as well as in chemotherapy-refractory NSCLC patients treated with cetuximab and avelumab. 16S rRNA sequencing revealed that long-term responding patients (with progression-free survival of 9-24 months) experienced significant increases in two butyrate-producing bacteria, *Agathobacter* and *Blautia*, compared to the patients with shorter progression-free survival (2-6 months) [117]. Significantly better progression-free survival was also observed in basal fecal samples, depending on whether or not these species were present [117].

Epithelial ovarian cancer, fallopian tube cancer, and primary peritoneal cancer, collectively referred to as ovarian cancer (OC), represent the deadliest gynecological malignancies [118]. Immunofluorescence of LPS and genetic testing methods, including 16S rRNA gene sequencing, Patho-Chip (pan-pathogen array), and 2bRAD sequencing, have advanced our understanding of the intratumor microbiome in OC [119-123]. *Propionibacterium acnes, Acetobacter, Chlamydia, Firmicutes, Proteobacteria*, and *Fusobacterium* have consistently increased, while *Lactococcus* has decreased in the upper female reproductive tract of PC patients and each of these microbes are believed to predispose someone to develop ovarian cancer [121, 124-127]. A high-diversity vaginal community state type refers to groups of vaginal microbiomes that can be identified by their types and amounts of *Lactobacillus* bacteria; this is linked to the presence and persistence of high-risk human papillomavirus (HPV), which leads to decreased cellular p53 activity and reduced T cell activity, making one more susceptible to OC [128, 129]. In a phase 2 immunotherapy clinical trial (NCT02853318) involving recurrent OC patients, researchers conducted an integrative multi-omics analysis to clarify the biological factors associated with a long-lasting positive clinical outcome [130]. Combining 16S RNA sequencing and metabolomics profiling of patient fecal samples, their research indicated that the fecal microbiome contributes to each patient’s unique biological environment influencing treatment response [130]. In responsive patients before and during treatment, researchers found the presence of *Ruminococcus* and *Leuconostoc mesenteroides*, which are known to generate lactic acid that can improve both helper and cytotoxic-T-cell activity [130]. This can enhance the effectiveness of chemotherapy and immunotherapy by boosting cytokine production [130-132]. They also noted a decrease in the metabolite indole, which helps balance the gut’s anti-inflammatory properties by enhancing T regulatory cell differentiation and suppressing the immune system through a diminished helper T cell response [130, 133, 134].

In breast cancer, various mechanisms allow the breast microbiota to regulate immune function and, in turn, influence tumor growth and development. Using 16S rRNA gene sequencing, a team of researchers characterized the microbiome of human breast tissue in more than 200 patients with breast cancer [135]. They found that bacterial strains vary in abundance based on breast tissue type, cancer stage, grade or degree of abnormality of cancer cells, histologic subtype, presence of estrogen and progesterone receptors, invasion of cancer into blood vessels and lymphatics, and whether the cancer has spread to the lymph nodes or not [135]. Interestingly, bacteria linked to benign tumors (*Anaerococcus, Caulobacter*, and *Streptococcus*) and the activation of T-cell responses (S*treptococcus* and *Propionibacterium*) were absent from breast cancer-associated tissue networks that maintain systemic homeostasis [135]. This suggests that the loss of these strains could intensify tumor growth by downregulating adaptive immune antitumor responses thus creating a pro-tumorigenic environment. Moreover, the downregulation of toll-like receptors (TLRs), which assist the body in recognizing pathogen associated molecular patterns (PAMPs) to trigger an immune response, was noted in breast cancer tissues [135]. A cross-sectional analysis of breast cancer survivors found differences in fecal microbial composition between those with and without obesity [136]. Survivors without obesity showed significantly higher relative abundances of *Ruminococcus, Streptococcus, Roseburia*, and *Dorea*, while survivors with obesity had a higher relative abundance of *Pseudomonas and Proteus* [136]. Triple-negative breast cancer (TNBC) is the most aggressive subtype of breast cancer. In patients with TNBC who achieved a pathological complete response (pCR)—meaning no signs of cancer were found in tissue samples after chemotherapy—*Bacteroides eggerthii* was consistently higher in the pCR group from the start of the study and continued to be elevated both before and after chemotherapy [137]. The serum and fecal metabolite profiles of TNBC patients were analyzed using liquid chromatography-mass spectrometry (LC-MS). The analysis revealed changes in carboxylic acids and benzene derivatives, as well as differences in the relative abundance of *Anaerococcus, Fischerella*, and *Schizosaccharomyces* at the genus level, which have previously been associated with breast cancer [138]. Carboxylic acids include amino acids (AAs) and their derivatives, Tricarboxylic acid (TAC) cycle metabolites, SCFAs, medium-chain fatty acids (MCFAs), long-chain fatty acids (LCFAs), and bile acids. These compounds are widely found in living systems and are essential for immunity and metabolism, though they can negatively impact human health if dysregulated [139, 140]. Some studies on BC have focused on viruses, and tumors contained Cytomegalovirus and up to 50% of Epstein-Barr virus [141-143].

Recent studies associate disruptions to the balance of the gut microbiome with the advancement and prognosis of prostate cancer (PCa), which remains the second most frequent male malignancy worldwide [144]. The microbiome profile of a tumor, peri-tumor, and nontumor prostate tissues was assessed in 16 radical prostatectomy specimens using massive ultradeep pyrosequencing. It revealed *Propionibacterium* species as the most abundant bacteria across all groups and an overrepresentation of *Staphylococcus* species in the tumor and peri-tumor tissues [145]. A systematic review and meta-analysis conducted across multiple databases exposed that patients with PCa has significantly less species diversity in their gut microbiome than the control group [146]. The analysis also showed that the proportion of *Proteobacteria, Bacteroidia, Clostridia, Bacteroidales, Clostridiales, Prevotellaceae, Lachnospiraceae, Prevotella, Escherichia-Shigella, Faecalibacterium*, and *Bacteroides* species was higher in prostate PCa patients [146]. In the control group, the proportion of *Actinobacteria, Bacteroidetes, Firmicutes, Selenomonadales, Veillonella*, and *Megasphaera* species was higher than the PCa patients [146]. Prostate cancer cells are directly influenced by contact with microbiota from nearby tissues and urine. Basic research indicates that the gut microbiome plays a crucial role in the underlying biology of prostate cancer through the activity of metabolic products derived from microbes [147]. In the Prostate, Lung, Colorectal, and Ovarian Cancer Screening trial, mass spectrometry was performed on baseline serum samples collected from 173 cases of lethal prostate cancer and 519 controls. The data revealed that cases with the highest levels of choline, betaine, and phenylacetylglutamine—a gut microbiome metabolite with adrenergic activity—had a greater likelihood of developing lethal prostate cancer [37]. New evidence implicates the gut microbiota in weakening urologic health via increased intestinal permeability [148]. The Carbohydrate and Prostate Study 2 (CAPS2) clinical trial showed that a low-carbohydrate diet aided in weight loss, which significantly improved intestinal permeability in prostate cancer patients [149]. This improvement correlated with a slowdown in cancer progression, as indicated by the prostate-specific antigen (PSA) doubling time—a measure of how quickly PSA levels rise in the blood. A doubling time longer than 6 months is a positive predictor, indicating a lower likelihood of aggressive treatment being necessary in the future and improved survival rates. Additionally, the more significant the weight loss, the longer the PSA doubling time tends to be [149]. Oral administration of an antibiotic mixture along with a high-fat diet that included a substantial amount of lard in a mouse model for prostate cancer resulted in an increase in *Rikenellaceae* and *Clostridiales*, inhibited prostate cancer cell proliferation, and reduced prostate Igf1 expression as well as circulating insulin-like growth factor-1 (IGF1) levels, which are known to directly promote the proliferation of prostate cancer cell lines [150]. Studies exploring the influence of diet and exercise in men with high-risk localized prostate cancer who are receiving androgen deprivation therapy (ADT) found that exercise, regardless of diet, led to positive changes in gut microbial diversity and composition, along with a corresponding decrease in indole-3-carboxaldehyde, which regulates mucosal reactivity and inflammation [40, 151].

### (ii) CANCER TREATMENT RESPONSE AND THE GUT MICROBIOME

**Figure 3.**
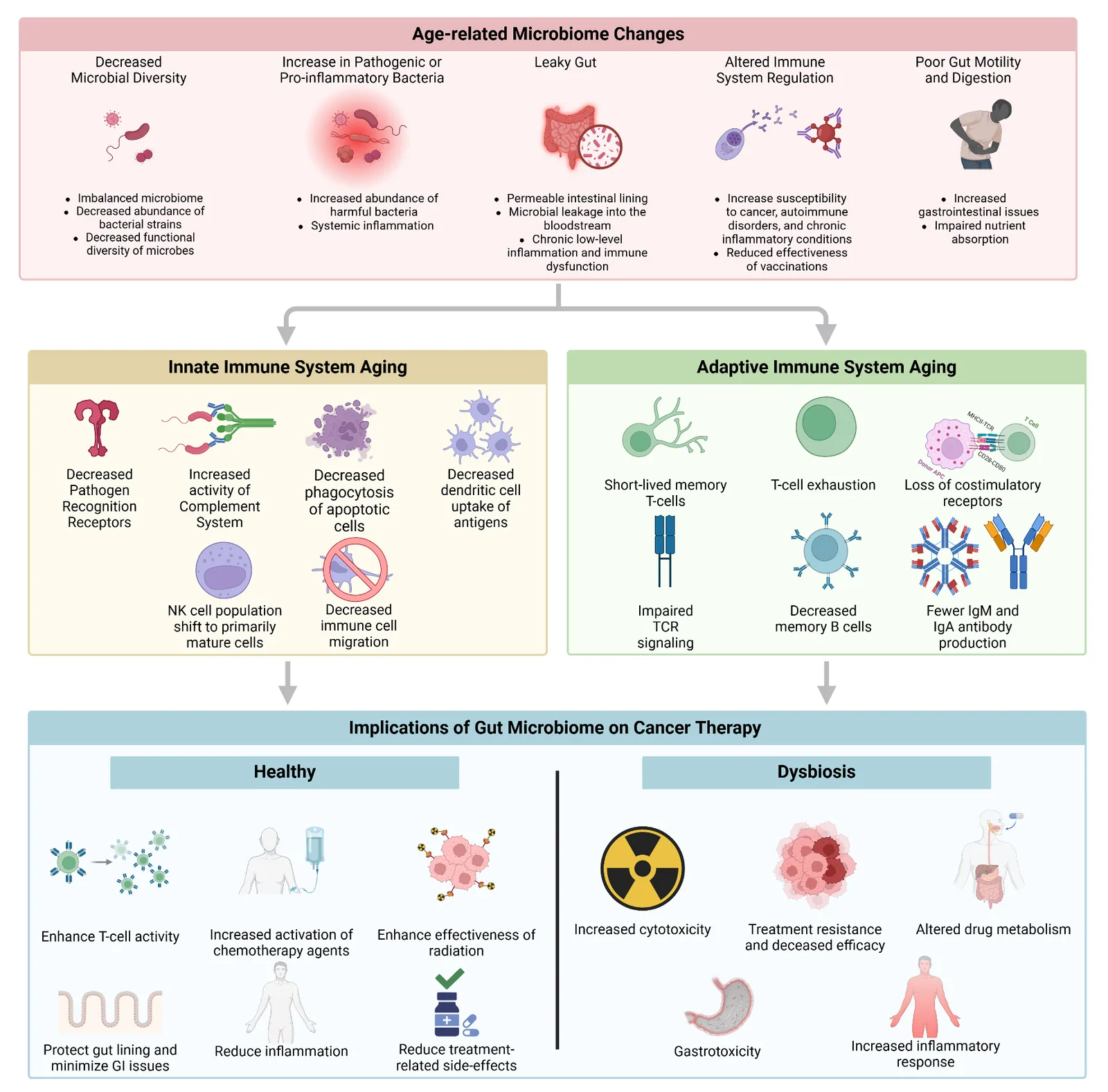
**The Gut Microbiome in Cancer Therapy Treatment Outcomes**. As individuals age, the gut microbiome experiences increased inflammation, greater permeability of the intestinal lining, impaired immune function, poor digestion, and a decline in microbial diversity regarding functional activity, types of microbial species, and overall abundance of microbes. Moreover, aging is associated with weakened innate and adaptive immune responses and an increased susceptibility to infections. In age-related cancers, dysbiosis combined with an aging immune system can result in ineffective cancer treatment and increased toxicity or adverse side effects.

Alterations in the gut microbiome contribute to the growth and development of age-related cancers and play a pivotal role in the response to cancer treatment (Fig. 3). Aging significantly shapes the gut microbiome, which in turn, impacts the growth and development of age-related cancers and influences cancer treatment outcomes. Emerging data reveal that the gut microbiome can both positively and negatively affect the efficacy and side effects of cancer therapies [152]. Cancer therapy encompasses surgical therapy, chemotherapy, targeted therapy, radiation therapy, hormone therapy, stem cell therapy, and immunotherapy. Chemotherapy, radiotherapy, and immunotherapy are the treatments most documented to be affected by the gut microbiome [152-163]. Chemotherapy operates systemically and targets rapidly dividing cells to control or eliminate cancer from the body [164]. Radiotherapy can be external, internal, or systemic, and it aims to induce DNA breaks in rapidly dividing cells to shrink early, recurring, or late-stage cancers [165]. Cancer immunotherapy harnesses the host’s own immune system to ideally differentiate normal and cancerous cells, to be potent enough to trigger immune clearance of many tumor cells, and lastly, to be durable enough to thwart reemerging tumors [166]. Cancer immunotherapy stands out as a revolutionary advancement in recent years, including immune checkpoint therapy, exemplified by programmed cell death 1 (PD-1) and cytotoxic T lymphocyte-associated antigen 4 (CTLA-4) inhibitors, as well as adoptive T-cell therapy (ACT), represented by chimeric antigen receptor T cell (CAR-T) therapy and cancer vaccines. Although cancer immunotherapy occupies a crucial role as a treatment option, many patients receive immunotherapy with limited benefit, mainly due to primary or acquired resistance, especially in individuals with melanoma and non-small cell lung cancer. Several studies have indicated that gut microbiota may contribute to this disparity in efficacy [167].

#### Aging Immune System

Natural immunity consists of three interconnected components: (i) physiological barriers, (ii) innate immunity, and (iii) adaptive immunity, all of which can be influenced by aging [168]. Aging is linked to immunosenescence, which is defined as a decline in immune system function as an individual ages [169, 170]. Elderly people commonly show dysregulated immune responses to pathogens, decreased effectiveness of vaccinations, and increased susceptibility to various diseases, including cancer, autoimmune disorders, and chronic inflammatory conditions [170, 171]. The innate immune system functions globally through cytokine signaling, peroxide and nitric oxide production, and the phagocytic activity of neutrophils, all of which decline with aging [170]. Natural killer (NK) cells undergo age-related population shifts from a less mature, cytokine-producing subset (CD14^+^CD56^dim^) to a mature subset (CD14^+^CD56^bright^) [172, 173]. However, both subsets show decreased activity and migration and changes in the diversity of their activating and inhibitory receptors [172]. The innate immune system recognizes the PAMPs of microorganisms through pattern-recognition receptors (PRRs), such as nucleotide-binding oligomerization domain-like receptors (NLRs), C-type lectin receptors (CLRs), RIG-1 like receptors (RLRs), C-reactive proteins (CRPs), and TLRs [88, 174-176]. However, these receptors are reportedly diminished in aged neutrophils [177]. Elevated CRP activity is a biomarker of aging and is associated with inflammation and fibrosis [178]. The involvement of the complement system in the pathogenesis of age-dependent diseases and their complications, such as age-related macular degeneration and type 2 diabetes, has been established [179, 180]. Similarly, macrophages and their capacity to phagocytose apoptotic cells is somewhat reduced with aging [181]. As people age, changes happen in the gut microbiota, including reduced overall diversity and a higher prevalence of pro-inflammatory species. These alterations can lead to systemic inflammation and a range of age-related diseases [65, 182, 183]. The clinical consequences of adaptive immune aging are significant. Initial signs of declining immune competence appear around 50 years of age, with increasing clinical impact in the 70s to 90s [184]. Key features of an aging T-cell system include difficulties in T-cell maintenance, compromised DNA integrity, short-lived effector memory T cells, exhausted T cells, a reduction in stem-like memory T cells, a decrease in tissue-residing T memory cells, epigenetic changes, loss of immune cell surface markers, and impaired T-cell receptor signaling [184]. Memory T cells without prior antigen exposure also increase with age [185]. This results in diminished T-cell responses, reduced generation of cytotoxic T-cells in response to new antigen encounters, chronic inflammation, cellular dysfunction, replicative stress, decreased T-cell survival and lowered proliferation. Aging is also linked to a significant decrease in CD27 dull memory B cells, a population that connects innate and adaptive immune functions. Age-related changes in dendritic cells result in decreased uptake of antigens and/or microbes, which leads to reduced maturation, characterized by diminished migration and expression of costimulatory molecules (e.g. CD27 and CD28) and key cytokines critical for T cell stimulation (e.g. IFN-α, IL-12) [186]. B cells from older individuals produced markedly fewer IgM and IgA antibodies than younger individuals [187]. This indicates that the immune system of older adults is well-equipped to respond to frequently encountered antigens but has a limited capacity to react to new pathogens.

#### Aging Immune System and Cancer

An aging immune system significantly impacts age-related cancer development and treatment outcomes. Under normal circumstances, the immune system can prevent tumor formation through a process known as "immunosurveillance" [188]. However, a key aspect of tumor development involves cancers acquiring immune escape mechanisms, such as inducing or recruiting immunosuppressive cells (e.g., regulatory T cells, myeloid-derived suppressor cells, and tumor-associated macrophages) and increasing the expression of various immunosuppressive molecules (e.g., PD-1 and PD-L1) [189, 190]. The connection between aging, cancer, and decreased protective immunity is well-established [191]. As the adaptive immune system ages, it experiences immune senescence, resulting in deficiencies in both humoral and cellular components, notably, diminished T cell function [191-193]. Three key T cell functions are particularly affected by aging, including (i) a decline in the number of naïve T cells due to diminished thymic output, (ii) an increase in the memory T cell subset characterized by elevated type I/type II cytokine production profiles, and (iii) the accumulation of terminally differentiated T cells (cells are committed to a particular function and can no longer divide) that are dysfunctional and exhibit limited T-cell receptor (TCR) repertoire diversity [192, 194, 195]. These terminally differentiated T cells are mainly CD8+ T cells that arise from chronic exposure to viral infections, such as cytomegalovirus, leading to longitudinal expansion of groups of CD8^+^ T cells, a phenomenon known as 'memory inflation’ [196]. Furthermore, the senescence of these T cell subsets may stem from the limits of clonal expansion, potentially associated with telomere erosion [197-199]. Senescent T cells exhibit low telomerase activity, short telomeres, and DNA damage (e.g. γH2AX foci), leading to apoptosis resistance and increased β-galactosidase activity which bind to immune cells to trigger a response [186]. Additionally, these cells diminish the expression of costimulatory molecules CD27 and CD28 while increasing terminal differentiation markers such as killer-cell lectin-like receptor G1 (KLRG1) [198]. Regulatory T cells (Tregs) undergo more severe senescence than effector T cells as they age. This process involves the downregulation of DDB1- and CUL4-associated factor-1 (DCAF1), which impacts the regulation of reactive oxygen species (ROS) levels via glutathione S-transferase (GSTP1), resulting in increased proliferation and activity of aged Tregs [200].

#### Immune Checkpoint Inhibitors (ICIs) and Role of Gut Microbiota in ICI Response

Immune checkpoint inhibitors (ICIs) significantly advance cancer immunotherapy [201]. ICIs are a form of immunotherapy that targets upregulated receptors on T cells, reactivating the immune response [202, 203]. The most common co-inhibitory receptors ICIs target are (PD-1 and CTLA-4. Several ICIs have been approved or are undergoing clinical trials for treating solid tumors, including melanoma, breast cancer, metastatic NSCLC, head and neck squamous cell carcinoma (HNSCC), and Merkel cell carcinoma [204-207]. However, the effectiveness of ICIs can be limited by host tissue immunity, with many responders eventually developing acquired resistance [201]. Recent studies suggest that gut microbiota composition may influence the response to ICIs since gut microbes and their metabolites can modulate host immunity, indirectly affecting treatment outcomes [208]. For instance, a study by Jin Y et al. found a strong correlation between the diversity of the gut microbiome and the response to anti-PD-1 immunotherapy in advanced NSCLC patients. Those with a favorable gut microbiome showed enhanced memory T cells and NK cells, indicating a stronger immune response capable of reducing the time needed to control tumor growth [209]. Furthermore, a U.S. clinical trial (NCT03829111) showed that combining *Clostridium butyricum* probiotics with dual checkpoint inhibitors (anti-PD-1 and anti-CTLA-4) enhanced response rates and progression-free survival in patients with advanced kidney cancer, indicating that probiotics may boost the effectiveness of immunotherapy. Similarly, research conducted at Jiangxi Provincial Cancer Hospital in China demonstrated that increasing beneficial bacteria improved PD-1 inhibitor efficacy in mice. This led to clinical trials that combined probiotics V9 (*Bifidobacterium lactis*) and M9 (*Lactobacillus rhamnosus*) with PD-1 inhibitors in patients with non-small cell lung and liver cancer (NCT05094167, NCT05032014) to assess the outcomes of the combined therapy against monotherapy [209]. However, *Bacteroides* species have mixed effects because they can promote the expansion of T-reg cells and stimulate the production of anti-inflammatory cytokines, potentially weakening the immune response and hindering the body's ability to combat cancer [210]. Therefore, it is essential to consider these negative markers as they can affect patient outcomes.

#### Chemotherapy and Gut Microbiome Interaction

Chemotherapy is a treatment method that employs chemical substances, especially cytotoxic drugs, to destroy rapidly dividing cancer cells or hinder their growth. It is a fundamental aspect of cancer treatment, frequently used alone or alongside other therapies such as surgery, radiation, or immunotherapy [211]. However, the bidirectional relationship between the microbiome and chemotherapy is evidenced by the microbiome's ability to influence the metabolism and efficacy of cytotoxic chemotherapy, while chemotherapy-induced changes, in turn, alter the composition of the microbiome, further impacting treatment outcomes and patient responses [212]. A major side effect of chemotherapy treatment is gastrointestinal toxicity, primarily referring to mucositis, which can lead to infection, and diarrhea. Chemotherapy disrupts the microbiome and can cause gastrointestinal toxicity, which affects up to 80% of all patients receiving cancer treatment, leading to symptoms such as diarrhea, abdominal bleeding, and pain [213]. A report by Osterlund and colleagues proposed using *Lactobacillus* probiotics alongside chemotherapy to reduce gastrointestinal toxicity and successfully demonstrated protection against intestinal damage [214]. Additionally, it was found that *Lactobacillus* lessened the severity of mucositis and diarrhea caused by cancer treatment [215]. Since the 1960s, it has been understood that gut microbiota can metabolize drugs, including chemotherapeutic agents such as methotrexate (MTX), irinotecan (IRT), and several others [216]. Researchers have demonstrated that the use of antibiotics diminished the effects of the chemotherapy drugs, such as oxaliplatin and 5-fluorouracil (5-FU) in CRC [217, 218]. Bawaneh et al. demonstrated that enhanced populations of *Akkermansia muciniphila*, a mucin-degrading bacterium commonly found in the human gut, favor the efficacy of the well-known chemotherapy medication Doxorubicin (DOX) [219]. While the beneficial role of bacteria is found for some species, others exacerbate the deleterious effects of chemotherapy. In a report by Yu and colleagues, they demonstrated that *Fusobacterium nucleatum* reduces the efficacy of oxaliplatin and 5-FU in CRC by disrupting autophagy and is associated with patients experiencing recurrence after chemotherapy [111]. Growing evidence suggests that bacterial enzymes can deaminate the chemotherapeutic agent gemcitabine (GEM) into an inactive form. Geller et al. demonstrated that gamma-proteobacteria promoted GEM resistance in pancreatic cancer [220]. Additionally, a report by Vande et al. showed that *Mycoplasma hyorhinis* enhanced GEM resistance in breast cancer [221].

#### Radiotherapy and Gut Microbiome Interaction

More than 50% of cancer patients need radiotherapy as part of their treatment, which works by delivering high-energy ionizing radiation to destroy malignant cells [222, 223]. Despite reports that the gut microbiota can have both positive and negative effects on the effectiveness of radiotherapy, the immediate impact of gut microbiota on this treatment method remains deeply misunderstood. A recent study by Crawford and Gordon found that the gut microbiota increased the radiosensitivity of lymphocytes and endothelial cells in the mesenchymal core of small intestinal villi in sterile mice that underwent whole-body irradiation [224]. In CRC tissues and cells, high expression of the oncogene FOXQ1 has been observed, along with enhanced nuclear translocation and β-catenin expression, which are mediated by SIRT1 up-regulation and are beneficial to intestinal pathogenic micro-organisms linked to CRC. The positive correlation between increased radioresistance in CRC cells and poor prognoses in CRC patients indicates an interconnected relationship between gut microbiota and radioresistance [225]. Another example is the fast-induced adipokine (Fiaf), produced by the small intestine's villous epithelium. Fiaf can be suppressed by gut microbiota, leading to increased fatty acid accumulation in adipose tissues and the liver. Recent studies have shown that a reduction in Fiaf can result in a loss of resistance to radiation-induced apoptosis in villous endothelial cells and lymphocytes [224, 226]. Radiation can trigger an antitumor immune response by priming tumor-associated antigens, cytotoxic CD8+ T cells, and the abscopal effect or the shrinkage of untreated tumors concurrently with shrinkage of tumors within the local treatment area [227] [71].

In summary, the interaction between gut microbiota and cancer treatments—such as immunotherapy, chemotherapy, and radiotherapy—highlights microbes' complex role in influencing treatment effectiveness and toxicity. The disorder of the gut microbiota disrupts biofilms, which may affect cancer progression through inflammation and immune regulation, signaling pathway activation, production of bacterial metabolites, intestinal metabolism, and other factors [228]. Further research into manipulating the gut microbiome could lead to personalized cancer therapies and improved patient outcomes.

### (iii) EPIGENETIC CHANGES AND AGE-RELATED CANCER

Epigenetics refers to changes in gene expression without altering the underlying genetic sequence. The most well-known epigenetic regulations include DNA methylation, histone modifications (such as acetylation, methylation, phosphorylation, and ubiquitination), and non-coding RNAs (ncRNAs) [229]. These mechanisms can affect various nuclear processes, including gene transcription and silencing, DNA replication and repair, and cell cycle events [230]. Epigenetics facilitates adaptation to dynamic environmental changes, promoting a balance of biochemical processes that can lead to various diseases if disrupted [231]. Aging and cancer are interconnected and share common epigenetic alterations. As organisms age, there is widespread loss of histone proteins, chromatin remodeling, changes in histone modifications, and alterations in DNA methylation patterns throughout the genome. [232]. Changes in the epigenetic regulation of genes have been shown to enhance the expression of oncogenes, silence tumor suppressors, and ultimately promote tumorigenesis [233]. Such changes are valuable information for cancer risk prediction and diagnosis.

#### DNA Methylation and Cancer

DNA methylation involves constricting DNA structure and subsequent blockage of transcription factors, resulting in decreased gene expression. This modification is primarily performed by the DNA methyltransferase (DNMT) family of enzymes [234]. Abnormal patterns of DNA methylation are a hallmark of nearly all human cancers [13]. In breast cancer, the upregulation of DNMTs correlates with poor prognosis due to the abnormal methylation of tumor suppressor genes, which promotes uncontrolled cell proliferation and results in metastasis, potentially serving as a diagnostic marker [235, 236]. For example, genes such as cyclin-dependent kinase inhibitor 2A (CDKN2A/^p16 INK4A^) and retinoblastoma transcriptional corepressor 1 (RB1) [83], as well as promoter genes that affect telomerase activity [84], are affected by methylation. The expression of the tumor suppressor gene superoxide dismutase 3 (SOD3) and glutathione S-transferase Mu 2 (GSTM2) has been implicated in aggressive, high-grade breast tumors, where promoter hypermethylation is associated with poor survival in patients [237]. Similarly, advanced prostate cancer involves aberrant DNA methylation at cytosine bases located in a CpG dinucleotide due to increased DNMT expression, which is believed to be a key driver of prostate cancer lineage plasticity and histologic transformation [238-241]. A group of researchers inhibited DNMT expression in vivo using Decitabine and observed reduced tumor growth in patient-derived xenograft models of prostate cancer, along with increased expression of the costimulatory/coinhibitory immunoregulatory protein B7 homolog 3 (B7-H3), which is an emerging therapeutic target for cancer [242]. Epithelial ovarian cancer (EOC) is genetically susceptible, and there is increasing evidence that DNA methylation plays a role in the risk of developing it [243]. Studies of known EOC susceptibility genes suggest that hypermethylation of the BRCA1 promoter region increases the risk of developing familial and sporadic cancer [244-247]. The blood epigenome from ovarian cancer patients was profiled, revealing over 2,800 differentially methylated probes, with 71 genes consistently replicated across studies despite heterogeneity [243, 248]. The KRAS gene (Ki-ras2 Kirsten rat sarcoma viral oncogene homolog) encodes a GTPase transductor protein known as KRAS, which regulates cell division [249]. In colorectal cancer (CRC) cells that express active KRAS, the mitochondrial glutamate transporter SLC25A22 promotes the accumulation of the tricarboxylic acid (TCA) cycle intermediate succinate. This leads to increased DNA methylation, enhanced cell proliferation via downstream signaling of WNT/β-catenin, and greater resistance to cytotoxic chemotherapy medications used to treat cancer [250]. Researchers discovered that co-exposure to low-dose DNMT inhibitors (DNMTi) and inhibitors of the Enhancer of Zeste Homolog 2 (EZH2, a catalytic subunit of the histone methyltransferase Polycomb repressive complex 2 (PRC2)) sensitizes colon cancer cells [251]. This alters the epigenome, leading to the transcriptional up-regulation of the calcium-induced nuclear factor of activated T cells (NFAT) and activating protein 1 (AP-1) signaling pathway, which generates a productive immune response [251]. In patients with PDAC, hypomethylation of regulatory regions in the interferon-stimulated gene guanylate-binding protein 4 (GBP4) led to the gene's overexpression and T cell exhaustion, which was negatively associated with patient survival [252]. DNA-methylation sites in specific CpG-islands in tissue and circulating free DNA isolated from patient plasma have been used as a diagnostic biomarker tool to identify pancreatic ductal adenocarcinoma, lung, acute myeloid leukemia, gliomas, and esophageal adenocarcinoma [253-257].

#### Histone Modifications and Cancer

Unlike direct mutations to the DNA sequence, histone modifications are reversible, making them ideal targets for developing anticancer drugs. A wide variety of histone modifications have been identified and recognized for their vital role in regulating chromatin state, gene expression, and other nuclear processes [258]. Access to DNA by transcription factors and other elements is regulated by post-translational modification (PTM) proteins, such as histone methyltransferases (HMTs), which constrict chromatin structure, and histone acetyltransferases (HATs), which relax chromatin structure, making it more accessible for transcription. Characterization of drug-resistant breast cancer cells using flow cytometry, microarrays, PCR, and immunoblotting revealed alterations in apoptosis and cell-cycle profiles and the downregulation of histone genes H2A and H2B [259]. When these genes were targeted with histone deacetylase (HDAC) small-molecule inhibitors, the resistance was reversed, confirming that histone pathways are linked to drug resistance in breast cancer [259]. A clinical trial found that malignant breast tumors exhibited hypoacetylation of the histone mark H3K18ac and hypomethylation of H4K20me3, in contrast to benign breast tumors [260]. PRMT6, Protein arginine methyltransferase 6, methylates histone and non-histone proteins and has been shown to interact with the BRCA p21 promoter, leading to the inhibition of p21 expression and the regulation of cellular senescence [261]. Additionally, other histone methyltransferases, such as protein arginine methyltransferase 1 (PRMT1), coactivator-associated arginine methyltransferase 1 (CARM1), and PRMT6, have played a role in driving BRCA progression by modulating H3R2 methylation [262, 263]. Recently, PRMT6 has been found to promote breast cancer metastasis through asymmetrical di-methylation of the signal transducer and activator of transcription 3 (STAT3), which activates downstream signaling in the IL-6/STAT3 pathway, contributing to tumor metastasis and invasion [264]. PRMT1 is highly expressed in ovarian cancer and is known to facilitate the methylation of bromodomain-containing protein 4 (BRD4), which is crucial for both early embryonic development and cancer progression. This stimulates the inhibitory immune regulator TGF-beta and facilitates ovarian cancer invasion [265]. Pharmacological inhibition of PRMT1 has been demonstrated to reduce ovarian cancer proliferation, migration, and invasion both in vivo and in vitro [265]. In prostate cancer, primary prostate tumors can be differentiated from metastatic castration-resistant prostate cancer based on transcription factor binding and specific histone acetylation patterns (H3K27ac) at transcriptional enhancers [266]. In colorectal cancer, histone acetylation is associated with promoting an immunosuppressive tumor microenvironment. To address this, researchers combined the PD-1 monoclonal antibody, sintilimab, with the histone deacetylase inhibitor (HDACi), chidamide, and the anti-vascular endothelial growth factor (VEGF) monoclonal antibody, bevacizumab. This combination successfully enhanced cytotoxic T cell infiltration, resulting in a more immunologically active tumor microenvironment in patients with advanced disease [267]. Another clinical trial involving advanced NSCLC patients demonstrated that the administration of the DNMT inhibitor, decitabine, and the HDAC inhibitor, valproic acid, is effective in reactivating hypermethylated genes to reverse altered epigenetic programming observed in earlier stages of the disease [268]. Additionally, another HDAC inhibitor, entinostat, has shown anti-cancer activity in both in vitro and in vivo models of gastroenteropancreatic neuroendocrine tumors, where tumor growth significantly decreased by up to 68% of its original size since the beginning of the clinical trial [269].

#### MicroRNA Regulation and Cancer

The expression of DNA can also be altered epigenetically at the post-transcriptional level through mutations in miRNAs. Micro-RNAs regulate mRNA expression by binding to the untranslated region of the mRNA to target it for degradation or to temporarily silence the gene [270]. Epigenetic changes in miRNA can lead to uncontrolled gene expression that is typically downregulated and vice versa. Micro-RNAs and other epigenetic modulators can influence each other in a feedback loop, which can also become dysregulated [271]. It is important to note that a combination of different epigenetic modifications affects certain cancers. One example is prostate cancer, which is methylated at the GSTP1 gene, affecting detoxification and deacetylated at the human DAB2IP (hDAB2IP) gene, affecting signaling [272, 273]. In lung cancer, the microtubule-associated tumor suppressor 1 (MTUS1) gene is influenced by miR-19a and miR-19b, which are part of a miRNA cluster containing oncogenes. These miRNAs inhibit MTUS1, which then leads to lung cancer [274]. Epigenetic effects acting at various stages of the cell cycle are one factor that complicates the search for effective cancer therapies. Additionally, alterations in miRNA gene methylation have been found to contribute to the pathogenesis of ovarian cancer and breast cancer, where abnormal hypomethylation of oncogenic miRNAs is highly expressed, thus facilitating its growth and development [235, 275].

#### Environmental and Lifestyle Influences on Epigenetics

The exposome encompasses the cumulative environmental factors that influence an individual, including external conditions, lifestyle choices, and dietary habits [276]. Growing evidence links these exposures to the development of diseases by disrupting key epigenetic regulators of gene expression. Recent research suggests that external influences can cause changes such as DNA methylation, alterations in miRNA expression, and modifications of histones [277-279]. These changes can lead to increased inflammation, which is associated with a heightened risk of cancer. Furthermore, mutations in related proteins can disrupt chromatin structure, affecting the regulation of proto-oncogenes and tumor suppressor genes [280]. This dysregulation is critical to the onset and progression of various cancers, highlighting the importance of understanding how environmental and lifestyle factors impact health.

### Smoking

Epigenetic changes are crucial indicators of environmental exposure and disease susceptibility. Studies have linked tobacco smoking to significant alterations in DNA methylation at thousands of CpG sites. In a multi-omics study of type II alveolar cells from A/J mice exposed to environmental tobacco smoke, researchers discovered that this exposure resulted in notable changes in DNA methylation, hydroxymethylation, gene expression, and protein levels, leading to an inflammatory state that may promote oncogenic processes [281]. Additionally, cigarette smoke triggers the release of pro-inflammatory cytokines through NF-κB pathway activation, causing critical modifications to HDACs. This leads to significant shifts in gene markers on histones 3 and 4, impacting the regulation of the NOD-like receptor family pyrin domain containing 10 (NLRP10) molecules [282-284]. The evidence underscores the serious consequences of cigarette smoke on our epigenome.

#### High-fat diet

Obesogenic diets significantly accelerate cancer development through epigenetic mechanisms. Studies indicate that high-fat diets induce crucial epigenomic changes in metabolic and immune pathways that promote tumor growth. Alterations in histone modifications (such as histone H3K4me1 and H3K27ac along with histone H3 acetylation) and DNA methylation correlate with chromatin changes associated with the progression of breast, intestinal, and prostate cancers [285-287]. Furthermore, an increased inflammatory response following the consumption of high-calorie diets has been linked to changes in miRNA expression and DNA methylation processes. For instance, saturated fatty acids have been connected to DNA hypermethylation of the PPARγ1 gene promoter [288].

#### Air pollution and other environmental factors

Recent studies indicate that exposure to ambient air pollution, whether short-term or long-term, impacts epigenetic pathways associated with cancer development [289, 290]. For instance, Wang et al. found that long-term exposure to fine particulate matter (PM2.5) was linked to increased TNF-α expression due to a decrease in TNF-α gene methylation in women [291]. PM2.5 has been associated with an increased risk of mortality for various types of cancer, including those in the upper digestive tract, accessory digestive organs, breast cancer in females, and lung cancer in males [292]. Additionally, in a crossover trial, diesel exhaust influenced the expression of several miRNAs and mRNAs, leading to inflammatory cell recruitment and epithelial cell shedding, which can significantly heighten the risk of developing lung cancer [293]. Microplastics impact gene expression and inflammatory mediators by mimicking estrogen and androgen, triggering carcinogenesis, which leads to uncontrolled cell proliferation and increases the risk of cancers in the lungs, blood, breasts, prostate, and ovaries [294-296]. Epidemiological studies have indicated that exposure to Benzo(a)pyrene (BaP) is associated with alterations in methylation levels at 15 CpG sites. This occurs mainly through forming CpG- benzo(a)pyrene diolepoxide (BPDE) adducts, where the BaP metabolite BPDE binds to CpG, changing levels of 5-methylcytosine. BaP also inhibits DNA methyl-transferases and enhances the activity of HDACs, especially HDAC2 and HDAC3 [297]. This evidence highlights the significant influence of the environment on epigenetic regulation. Further investigation into the mechanisms by which environmental factors affect the epigenome is essential to advance our efforts to prevent cancer.

#### Therapeutic Implications of Epigenetic Changes

Due to their reversible nature, targeting epigenetic modifications has the potential to enhance cancer treatment responses. Currently, novel “epidrug” candidates have been identified in preclinical studies, and others are currently being tested in clinical trials. The development of epidrugs could provide multiple therapeutic functions, reduce therapy costs, and offer a new precision medicine mechanism to maximize treatment efficacy and minimize toxicity [298]. Epigenetic regulation through drug interventions shows great promise in modern cancer immunotherapy. Small molecule inhibitors of HDACs have proven to be toxic and difficult to produce. Still, strategies have been implemented to target HDAC activity to enhance the anti-cancer efficacy of certain drugs, such as HDAC-based dual-target inhibitors and PROTAC HDAC degraders [299]. A dual-target system benefits by addressing the limited efficacy of HDAC with small molecular regulation by targeting multiple pathways involved in tumorigenesis. PROTAC HDAC degraders are small molecules that selectively target HDACs and signal them for subsequent degradation. Another well-accepted anti-cancer drug is the poly (ADP-ribose) polymerase (PARP) inhibitor veliparib. This drug shows promise, but its efficacy is lacking after clinical trials. However, a group was able to demonstrate increased efficacy by combining veliparib treatment with HDAC inhibitors [300]. In the context of CRC, a phase 2 clinical trial utilizes a combination of treatments that target multiple signaling pathways, including the inhibition of HDACs. Rui-Hua Xu et al. summarize the results of combining an anti-PD-1 antibody, an HDAC inhibitor, and an anti-VEGF antibody to treat unresectable, chemotherapy-refractory, locally advanced or metastatic microsatellite stable/proficient mismatch repair (MSS/pMMR) colorectal cancer [267]. They demonstrated that this triple treatment increased CD8+ T cell infiltration, positively enhancing the immune response in the tumor microenvironment. Epigenetic alterations—including DNA methylation, histone modifications, and miRNA dysregulation—play a central role in cancer development and progression. Environmental and lifestyle factors further influence these changes, contributing to disease risk. Advances in understanding epigenetic mechanisms have paved the way for targeted therapies and precision medicine, offering hope for improved cancer treatment prevention.

### (iv) EPIGENETIC CHANGES BY GUT MICROBIOME

**Figure 4.**
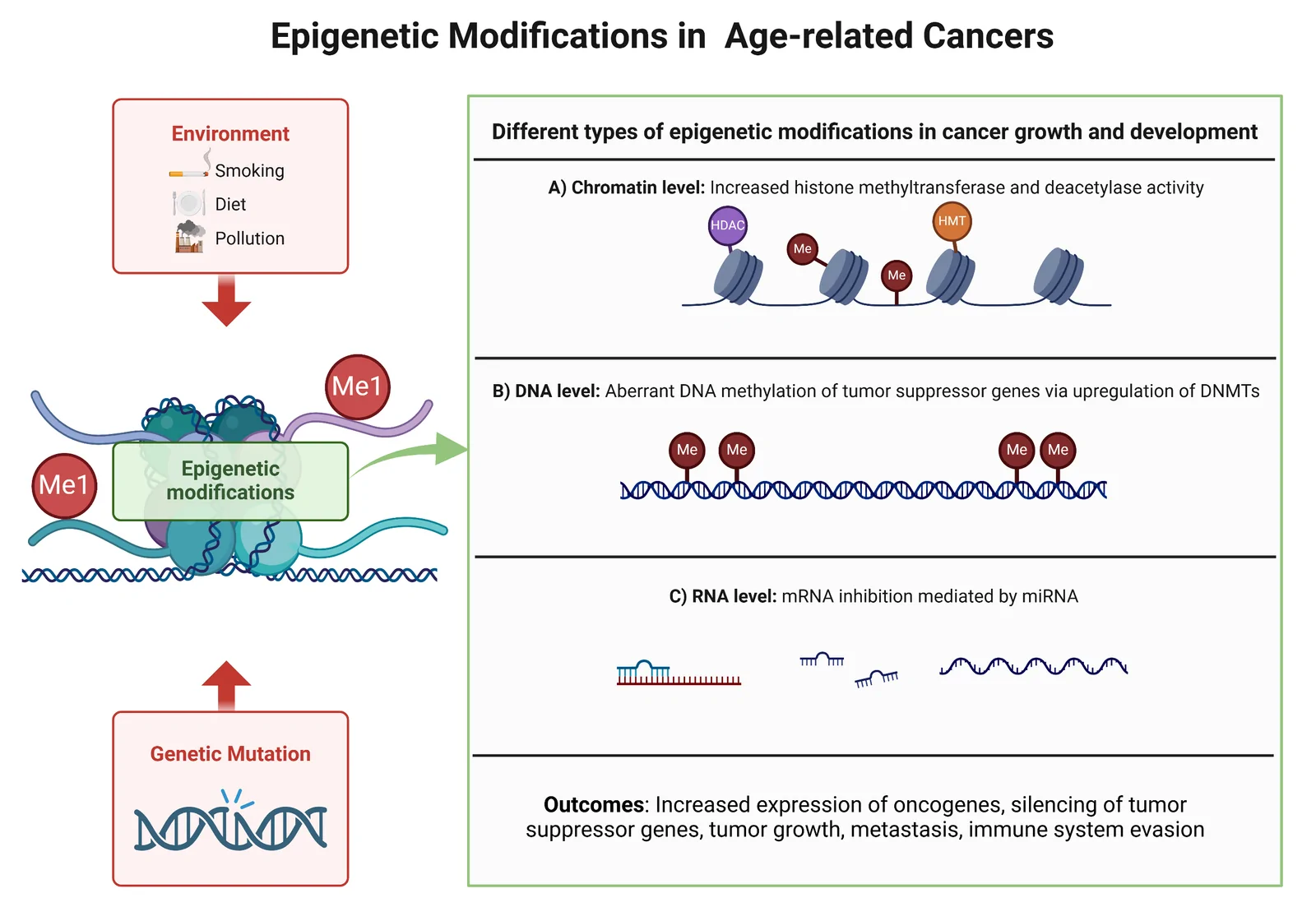
**Epigenetic Modifications in Age-related Cancers**. Age-related cancers are partly driven and worsened by genetic mutations and harmful environmental exposures, including smoking, a high-fat diet, and pollution. In response, the body alters its epigenetic landscape to silence tumor suppressor genes and promote tumor growth through the actions of HDACs, HMTs, DNMTs, and miRNA.

The complex interaction between gut microbiota and the surrounding tissue in the gut contributes to epigenetic changes (Fig. 4) in key regulatory processes across various organ systems, which, when dysregulated, can promote and drive diseases such as cancer [301-303].

#### Mechanisms of Epigenetic Regulation by Gut Microbiota

A range of specific gut bacteria have been linked to the regulation of the cancer environment and metastasis [111, 112, 304-306]. Seven different gut bacteria, including the *Fusobacterium nucleatum, Treponema medium, Peptostreptococcus stomatis, Gemella morbillorum, Catonella morbi, Peptoanaerobacter yurli*, and *Peptococcus simiae*, showed significant enrichment in DNA CpG methylation in tumor microenvironments which lead to substantial gene expression changes in tumor-suppressing pathways [307, 308]. The *Fusobacterium nucleatum* and other oral bacteria, such as *Treponema denticola*, have also been known to promote cancer progression and aggressivity via modulating crosstalk between TLR/MyD88 and integrin/FAK signaling pathways [307]. The *F. nucleatum* dysbiosis in laryngeal microbiota has been implicated in developing some forms of head and neck squamous cell carcinoma and esophageal cancer [309]. Where in the case of esophageal cancer, long interspersed nuclear element-1 (LINE-1) hypomethylation (a measure for unfavorable clinical outcomes) is shown to be correlated to *F. nucleatum* presence in the gut microbiome [310]. Also, it has been demonstrated that intracellular *F. nucleatum* infection mediates m6A methylation in a METTL3-mediated manner that promotes the metastasis of esophageal squamous cell carcinoma [311]. This evidence suggests a strong correlation between specific microbiota, such as *F. nucleatum*, and the epigenetic changes seen in various cancers, such as colorectal, esophageal, and laryngeal that all have proximity with these bacteria. However, the vast diversity of bacteria in the gut suggests that many important interactions and bacterial species have yet to be identified. More research is needed to fully understand the microbiota-body axis that contributes not only to homeostasis but also to the onset and progression of disease. In CRC, human intestinal epithelial cells were exposed to *Lactobacillus. acidophilus, Bifidobacterium infantis*, and *Klebsiella* species exhibited differential methylation changes in 200 regions of DNA [312]. Various studies on mouse models demonstrated that dysbiosis contributed to oncogenic epigenetic signatures not present in normal intestinal tissue, as evidenced by the hypermethylation of transcriptional promoters including SFRP1, 2, 3, PENK, NPY, ALX4, SEPT9, and WIF1, along with alterations in the methylation status of genes increasing with age [313, 314]. Intestinal *Akkermansia muciniphila* can predict the response to PD-1 blockade, a treatment that aims to enable T cells to eliminate tumor cells [315]. Further studies investigating the mechanisms behind A. *muciniphila*-dependent regulation of the host tumor microenvironment have identified ways bacteria can directly alter the host epigenome. A study by Zhang et al. demonstrated that *A. muciniphila* directly modified host proteins through extracellular vesicle release and subsequent uptake by the host via micropinocytosis, resulting in histone 3 acetylation on Lys14 (H3K14ac) [316]. This acetylation subsequently led to immune activation through the transcriptional activation of HSP70, which activated immune response elements and halted tumorigenesis [316]. Aging-related shifts in gut microbiome composition are influenced by diet and environmental factors. These shifts are linked to health outcomes and disease, with specific taxa, such as *A.muciniphila*, demonstrating the ability to modulate the host's immune and epigenetic landscape. This underscores their potential role in maintaining health and shaping therapeutic responses, particularly in cancer immunotherapy.

#### Microbe Metabolic Products and Their Role in Epigenetic Changes

Microorganisms in the gut microbiome can produce bioactive substances, such as folates, short-chain fatty acids (butyrate and acetate), biotin, and others, which help facilitate epigenetic modifications and potentially drive disease progression [317]. For instance, butyrate can activate epigenetically silenced genes in cancer cells, like p21 and BAK [318]. *Lactobacillus plantarum* and its metabolite, indole-3-lactic acid, have been shown to relieve intestinal inflammation, tumor growth, and gut microbiome imbalance by stimulating the production of the cytokine IL-12a in antigen-presenting cells [319]. This occurs by strengthening H3K27ac binding at enhancer regions, which activates the cytotoxic T-cell immune response against tumor growth [319]. Microbiota-derived inositol phosphate also increases HDAC3 activity in the intestine, and commensal bacteria (e.g., *Escherichia coli*) stimulate HDAC activity through the metabolism of phytate and the production of inositol-1,4,5-trisphosphate (InsP3), promoting resilience and swift recovery following intestinal damage [320]. According to Tang et al., the gut microbiota regulates the expression of genes related to hepatic lipid metabolism by altering histone modifications, including acetylation and methylation [321]. Gut microbiota can sustain intestinal immunological homeostasis by epigenetically regulating immune pathways, such as the methylation of TLR4, through the involvement of DNA methyltransferase 3 [322]. One regulatory factor affecting this relationship is epigenetic modulation, which regulates gene expression without altering the DNA sequence [94]. These modifications include DNA methylation and histone modifications, which are crucial in maintaining cellular functions and can be influenced by specific microbial metabolites [323]. Mechanistically, microbiota releases bioactive compounds such as SCFAs, with significant forms including acetate, propionate, and butyrate, which act as inhibitors of HDACs [324]. As a microbial metabolite, SCFAs play a crucial role in modulating immune responses by influencing the function, differentiation, and proliferation of immune cells through changes in gene expression via epigenetic modulation [324, 325]. This regulation extends beyond surrounding cells and has influenced diseases such as diabetes, cardiovascular disease, pulmonary disease, and cancer [302, 321, 326]. Butyrate-dependent epigenetic regulation in the gut has been implicated in obesity, diabetes, cardiovascular disease, pulmonary diseases, and cancer [327-329]. SCFAs function as anti-inflammatory and have been reported to alter pro-inflammatory cytokines TNF- α IL-6 through alterations of HDAC expression in monocytes and adipose tissue macrophages [14, 140, 330]. Other microbially derived macromolecules, such as exopolysaccharides, can also downregulate gut inflammation [331, 332]. Intestinal microbes are also involved in the absorption and excretion of minerals, including zinc, iodine, selenium, and cobalt, and they participate in epigenetic processes [333]. Several enzymes that modify chromatin epigenetically, such as methyltransferases, acetyltransferases, deacetylases, Bir A ligase, phosphotransferases, kinases, and synthetases, are derived from the intestinal microbiota [334]. All these interactions suggest a complex gut-body axis in which the gut and microbiota tightly regulate each other. Changes in epigenetic modulation of key regulatory genes alter gene and protein expression; many of these genes and pathways have been implicated in various age-related cancers, suggesting a strong correlation between tumor environments and gut microbiome regulation [95, 106, 107] [150, 323, 326, 335, 336].

### (v) POLYPHARMACY AND ALTERNATIVE THERAPY

Aging is linked to various diseases, especially chronic conditions, which increase the likelihood of being prescribed multiple medications [5]. Polypharmacy refers to the concurrent use of different drugs to address complex, multifaceted diseases and is often considered a geriatric condition [9, 337]. Individuals exposed to polypharmacy face the risk of taking medications for prolonged periods, at incorrect frequencies, without achieving their intended effects [337]. They may also continue redundant medications, exposing them to risks such as adverse drug reactions, drug-disease interactions, and drug interactions, in addition to increased financial strains and higher healthcare expenses [10, 337]. All these risks can be summarized by the term potential inappropriate medication (PIM) [337]. PIM has been identified as a significant risk factor for the development of adverse drug events in older adults [338]. Older individuals with a predisposition to frailty and faster deterioration due to their inability to functionally compensate are at a higher risk of experiencing adverse drug events [337]. Patients with cancer are often prescribed numerous medications for the disease itself and for supportive care. Consequently, these patients face a high risk of medication-related incidents [10, 11]. Multi-target drugs and polypharmacy ideally combine underexplored therapeutic actions with clinically established mechanisms to enhance efficacy, alleviate side effects, accelerate the onset of action, and treat a broader range of symptoms. Considering the relationships among aging, cancer, and polypharmacy, these three concepts are closely intertwined, presenting an opportunity to study these parameters in further conjunction.

Epigenetic modifications are promising targets for polypharmacy strategies because they allow for the simultaneous targeting of multiple pathways. This approach can effectively address a broader spectrum of symptoms and diseases, particularly those with complex genetic and environmental interactions, such as cancer, neurodegenerative disorders, and cardiovascular diseases [339-341]. Incorporating these polypharmaceutical techniques in drug administration and research, deep phenotyping, and the construction of pharmacological interactomes is essential for understanding the genetic basis of drug responses [342]. This allows researchers to identify new therapeutic targets and optimize drug regimens to minimize adverse effects while enhancing efficacy. Epigenetic mechanisms in disease are an increasingly studied field where integrating pharmacogenomics with polypharmacy offers a new avenue for personalized medicine in various diseases, including Alzheimer’s disease (AD), Parkinson’s disease (PD), and several age-related cancers [343].

Genetic polymorphisms in genes involved in drug metabolism processes have been demonstrated [344]. Histone acetylation and methylation are attractive substrates in new multi-target and polypharmacy strategies [345]. Rapidly accumulating data from genomics and other omics analyses enable the construction of pharmacological "interactome" networks, acting as regulators or key factors in various biological processes, particularly drug absorption, distribution, metabolism, transport, and excretion. These networks synergize to maintain optimal drug dosage and duration at therapeutic targets and may lead to drug-drug interactions (DDI) [343]. Thus, deep phenotyping and interactome networks are particularly important in applying rational polypharmacy. Cardiovascular diseases, AD, neurodegenerative disorders (NDD), atherosclerosis, myocardial infarction, and depression have complex genetic backgrounds and substantial biological diversity due to various genetic, metabolic, and environmental factors [346]. They cannot be attributed to a single gene variant alone [347]. These diseases are more likely caused by the combined effects of multiple phenotype-related genes and environmental factors. The complexity of phenotypes in cardiovascular diseases, NDD, AD, PD, and other disorders highlights the fact that no single therapy can cure such complex diseases. At this point, the idea of targeting multiple targets simultaneously - polypharmacy becomes particularly important.

### (vi) GUT-ORGAN AXIS

Everyone ages, but not everyone ages the same way. This variation stems from biological changes in the gut microbiome that may be linked to various illnesses. Substantial evidence indicates that the human gut microbiome can establish a functional relationship with organs beyond the gut. The term “axis” refers to the different pathways through which one part of the body can communicate biochemically with another. The gut-body axis highlights the complex regulatory network in which the influence of gut microbiota maintains intestinal homeostasis while also impacting distant organs and systems. A key driving force in this process is epigenetic modulation influenced by microbial metabolites, which vigorously promote healthy states but can also trigger the onset of diseases. This emphasizes the potential for therapeutic strategies targeting the gut microbiome and the epigenetic modifications associated with disease states. To address these epigenetic changes caused by microbiota, the field of polypharmacy aims to enhance therapeutic efficacy in managing the complex nature of the gut-body axis. Overall, pharmacogenomics contributes to understanding the genetic basis of drug responses, thus assisting in identifying new therapeutic targets and molecular biomarkers to evaluate treatment efficacy.

Furthermore, gut microbiota is crucial in regulating the immune system and influencing brain function by affecting systemic inflammation [71]. The gut-brain axis (GBA) refers to the two-way communication between the gastrointestinal tract (gut) and the central nervous system (brain). It is essential in maintaining homeostasis among the gastrointestinal, central nervous, and microbial systems. Thus, the gut, brain, and microbiome are the three key players in this axis [347]. Chemotherapy-induced cognitive impairment (CICI) is a complication of chemotherapy treatment that leads to significant psychosocial challenges for cancer patients and survivors, including pain, fatigue, anxiety, depression, sleep disturbances, and cognitive dysfunction. Experiencing persistent and severe symptoms can lead to delays in cancer treatments, a decrease in tumor response, and a decline in a patient’s quality of life [348]. CICI has been undervalued by some healthcare professionals, causing cancer survivors to underreport and doubt the reality of their symptoms [349]. In the past, research has focused on the direct cytotoxic properties of anti-cancer agents. However, recent advances in the study of neurocognitive diseases have supported mechanisms based on neuroinflammation [350].

Consequently, the immunomodulatory properties of the gastrointestinal microbiota and its capacity to regulate neuroinflammation have gained increasing recognition as a factor believed to contribute to the underlying pathology of various neurocognitive conditions [350, 351]. Epidemiological data show correlations between negative gastrointestinal and neurological side effects in patients undergoing chemotherapy, suggesting an everyday molecular basis [134]. Experimental evidence has uncovered numerous potential communication pathways between the microbiota and the brain, including microbial-derived metabolites and their effects on the neural, hormonal, and immune-related signaling routes previously identified as the gut-brain axis [352, 353]. Neuroactive substances, such as 5-HT, γ-aminobutyric acid (GABA), and tryptophan metabolites, influence central nervous system activity and can be synthesized and released by the gut microbes. Microbes *Candida* and *Escherichia* utilize tryptophan in food to produce 5-HT, and *Bacillus* can produce dopamine [354, 355]. Microbiota-derived products, such as LPS, LPS-binding protein (LBP), peptidoglycan, and flagellin, are essential molecules that transmit information to the gut-brain axis. The pathogen recognition receptor, TLR4, is highly expressed on immune cells and detects LPS, consequently triggering its production.

**Figure 5.**
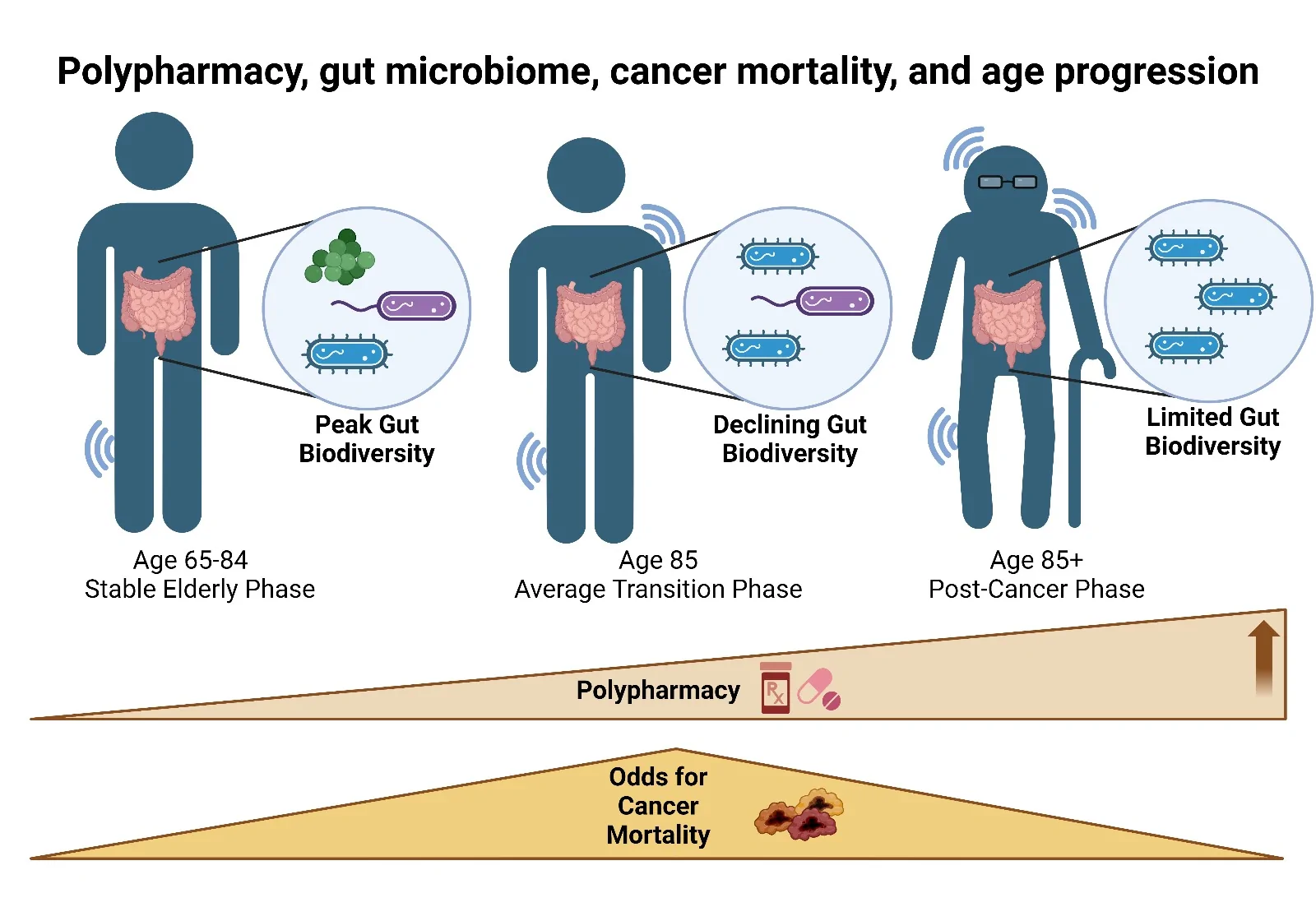
**Graphical illustration of how aging can impact the gut microbiome**. The aging population and the physiological changes that affect the composition and function of their gut microbiome can significantly influence the onset and progression of many illnesses, including cancer.

## TAKE AWAY MESSAGE

Age-related cancers are still poorly understood human pathologies associated with specific microbial changes and global shifts in microbiome community structure, often resulting from the concurrent use of multiple medications (Fig. 5). Therefore, understanding the various contributions of gut microbiota to carcinogenesis is expected to shed light on human variability in cancer development, progression, and treatment responsiveness. Currently, scientific evidence highlights the significance of epigenetic aging in tumorigenesis and its potential utility in predicting cancer risk. While many underlying mechanisms are likely specific to diseases or organs, investigating the microbiome offers great promise and represents the next frontier in medical research.
